# A Latent Class Analysis of Forensic Psychiatric Patients in Relation to Risk and Protective Factors

**DOI:** 10.3389/fpsyg.2021.695354

**Published:** 2021-07-20

**Authors:** Marija Janković, Erik Masthoff, Marinus Spreen, Peter de Looff, Stefan Bogaerts

**Affiliations:** ^1^Department of Developmental Psychology, Tilburg University, Tilburg, Netherlands; ^2^Fivoor Science and Treatment Innovation, Rotterdam, Netherlands; ^3^Department of Social Work, NHL Stenden University of Applied Sciences, Leeuwarden, Netherlands

**Keywords:** patient class, forensic patients, risk factors, protective factors, latent class analysis

## Abstract

Forensic psychiatric patients form a very heterogeneous population regarding psychopathology, criminal history, and risk factors for reoffending. Therefore, the present study aimed to investigate whether there are more homogeneous classes of forensic patients based on DSM-IV-TR Axis I and II diagnoses and previously committed offenses, by means of explorative latent class analysis (LCA). It was also investigated which risk and protective factors are significantly more prevalent in one class compared to other classes. The study sample contained 722 male forensic psychiatric patients who were unconditionally released between 2004 and 2014 from high-security forensic clinics. Data were retrospectively derived from electronic patient files. Five distinctive patient classes emerged: class with only Axis II diagnosis, class with multiple problems, antisocial class, psychotic class, and intellectually disabled class. These classes differed significantly in risk and protective factors. This study contributes to the understanding of patient classes and provides directions for future, class-tailored interventions.

## Introduction

In the Netherlands, individuals who committed violent crimes under the influence of a severe mental illness, personality disorder (PD), or deficits in cognitive development can be sentenced by the court to compulsory treatment to be carried out in a range of forensic psychiatric facilities. The aim of this forensic treatment is to protect society from dangerous offenders and to prepare them for successful reintegration into society (De Ruiter and Hildebrand, 2003; De Boer and Gerrits, 2007).

As prescribed by the Risk Need Responsivity model (RNR; Andrews and Bonta, 2006, 2010), effective treatment should focus more on targeting dynamic risk factors (i.e., “criminogenic needs”) that increase the likelihood of reoffending. In contrast, the Good Lives Model (GLM; Ward et al., 2007) places greater emphasis on protective factors, such as competencies and skills that can reduce the risk of recidivism and contribute positively during the rehabilitation of offenders. The RNR model comprises three principles: (1) the risk principle states that treatment intensity must be matched to the offender's level of risk for reoffending; (2) the need principle emphasizes the importance of assessing “criminogenic needs” [changeable (dynamic) risk factors directly related to recidivism] and targeting them in treatment; and (3) the responsivity principle implies that the intervention must match the motivation, learning style, and intellectual capabilities of the offender. According to the RNR model, the Big Four (i.e., history of antisocial behavior, antisocial personality pattern, antisocial cognition, and antisocial peers), and the Moderate Four risk factors (i.e., family/marital conditions, school/work, leisure/recreation, and substance abuse) are widely considered to be the most important predictors of reoffending (Andrews and Bonta, 2006, 2010).

On the contrary, protective factors have been less investigated (Schuringa et al., 2014; Serin et al., 2016), although there is some evidence that increasing offender's motivation and establishing a positive therapeutic alliance are crucial components of effective treatment, anchored in the principles of the GLM (Ward and Brown, 2004; Bogaerts et al., 2020). The GLM is a strength-based approach attempting to reduce the risk of reoffending by helping offenders living a more fulfilling and meaningful life (Ward and Brown, 2004). Nowadays, there is consensus that both risk and protective factors should be targeted during treatment (e.g., De Vries Robbé et al., 2015; Bogaerts et al., 2020). Gaining more insight into dynamic risk and protective factors is, therefore, the first and most important step in establishing effective offender treatment.

Although previous research has demonstrated certain beneficial effects of treatment in reducing the risk of reoffending, there remains considerable variation in the individual treatment outcomes in forensic patients (Gibbon et al., 2020; for a meta-analysis, see Gilling McIntosh et al., 2021; for a review, see Lipsey and Cullen, 2007; Smedslund et al., 2007). A plausible reason for this could be that forensic psychiatric patients form a very heterogeneous population with regard to type and severity of committed offenses, psychopathological characteristics, and risk and protective factors for reoffending (Van Nieuwenhuizen et al., 2011). For example, individuals with cluster B PDs often display clinical risk factors, such as impulsivity, addiction, and antisocial behavior, and are characterized by poor emotion regulation capacity and a lack of empathy (Kraus and Reynolds, 2001; Young et al., 2018; Jankovic et al., 2021). The latter is one of the most important predictors of serious and persistent criminal offending (Jolliffe and Murray, 2012), while poor self-regulation and higher impulsivity are considered to be crucial in explaining criminal behavior (Gottfredson and Hirschi, 1990). Likewise, untreated psychotic symptoms (e.g., paranoia) are important risk factors for violent behavior in psychotic patients (Bo et al., 2011). There is also empirical evidence for the association between specific risk factors and type of offense. In particular, lack of empathy (Hall and Hall, 2007; Jeandarme et al., 2017), deviant thoughts, impaired affect regulation (Scoones et al., 2012), and problem-solving deficits were found to be associated with sexual offenses (Bogaerts et al., 2004; Lockmuller et al., 2008). In addition, a history of substance use, hostility, and impulsivity were found to be associated with violent offenses (Craig et al., 2006; Jeandarme et al., 2017). Thus, patients residing in forensic psychiatric facilities may not respond equally to the treatment given, meaning they may have different treatment needs (Yiend et al., 2013; Van Der Veeken et al., 2017; Kip et al., 2018). Given this interdependence of psychopathology, type of offense, and risk and protective factors, identifying homogeneous classes of forensic psychiatric patients based on these characteristics could facilitate the development of more tailored and need-specific interventions in forensic correctional facilities. However, so far only a handful of studies have attempted to do so.

For example, Van Nieuwenhuizen et al. (2011) determined five patient classes based on psychopathology and type of offense among a representative group of 180 forensic patients. Similarly, Bogaerts and Spreen (2011) concluded that, based on risk and protective factors, three patient classes were sufficient to categorize a heterogeneous sample of 234 forensic patients with primary psychotic disorder and 348 forensic patients with PD. However, none of these studies considered psychopathology, type of offense, and risk and protective factors simultaneously. Based on previous research, Van Der Veeken et al. (2017) built on these earlier studies and examined classes in forensic psychiatric patients including all three domains. They identified four patient classes based on Axis I and II diagnoses according to the *Diagnostic and Statistical Manual of Mental Disorders*, 4th ed., text rev. (DSM-IV-TR); American Psychiatric Association (2000), type of offense, and risk factors derived from the Historical Clinical Future-30 (HKT-30; Comité Instrumentarium Forensische Psychiatrie, 2000) risk assessment tool. The first class, called the *antisocial class*, was characterized by cluster B PDs, and/or substance use disorders (SUD), various types of offenses such as homicide and maltreatment, and a few clinical high-risk factors including violations of terms, substance use, psychotic symptoms, and hostility. Likewise, the second class, called the *mixed class with multiple problems*, was characterized by cluster B PDs and comorbid psychotic disorder or comorbid SUD, and most patients in this class committed homicide or maltreatment offenses, similar to the antisocial class. It might be that the co-occurrence of psychotic disorders with cluster B diagnosis worsens the problematic behavior of the mixed class with multiple problems, compared to the antisocial class, which also encompassed patients suffering from cluster B PDs (Van Der Veeken et al., 2017). However, in contrast to the antisocial class, the mixed class with multiple problems displayed the highest levels of almost all clinical risk factors compared to the other classes. Some of these factors are violation of terms, problem awareness, impulsivity, hostility, and crime responsibility. The mixed class with multiple problems resembled the mixed cluster found by Bogaerts and Spreen (2011) and the patient with multiple problems found by Van Nieuwenhuizen et al. (2011). The third class, called the *maladaptive disordered affective class*, was characterized by pedophilic disorders and/or pervasive developmental disorders and/or PDs not otherwise specified (NOS), and this class was least likely to be characterized by SUD and/or cluster B diagnosis compared to the other classes. Most patients in this class committed homicide or a child sex offense, and had higher scores on clinical risk factors, such as lack of social skills, lack of empathy, crime responsibility, and problem awareness, relative to the other classes. This class was comparable to the class of patients with sexual problems and sexual crimes found by Van Nieuwenhuizen et al. (2011). Finally, the *psychotic first offender class*, the fourth class, was characterized by psychotic disorders, SUD, and/or cluster A, C, or NOS PDs and was less likely to be characterized by cluster B diagnosis in comparison to the other classes. Most patients in this class committed homicide offenses and had lower overall scores on risk factors compared to the other classes. The psychotic first offender class resembled the psychotic cluster found by Bogaerts and Spreen (2011) and the typical psychotic patient found by Van Nieuwenhuizen et al. (2011).

Although the study by Van Der Veeken et al. (2017) contributed to the understanding of the clinical profiles of forensic psychiatric patients and their characteristics, the risk assessment tools have been revised since the development of the HKT-30, in order to provide more accurate estimates of future risks. As far as we know, there is only one study investigating patient classes based on the clinical factors of the risk assessment tool—the Historical Clinical Future Revised (HKT-R; Spreen et al., 2014), and how these classes differ in comorbidity on Axis I, comorbidity on Axis II, type of drug and type of offense (violent vs. non-violent). However, this study was conducted on a smaller sample of 286 forensic psychiatric patients presenting with SUD (Schmitter et al., 2021), while at least 500 participants are required to reliably perform the latent class analysis (LCA) (Vermunt, 2004). Another shortcoming of the study by Schmitter et al. (2021) is that when examining comorbidity on Axis II, intellectual disability (ID) was not taken into account. Patients with this type of disability have rarely been studied in forensic settings and considering them in research would contribute significantly to the literature. Thus, to move the field forward, it is essential to study a more heterogeneous and diverse sample of forensic psychiatric patients, taking into account ID, in addition to clinical and PDs, and relying on the state-of-the-art risk assessment tools, such as the HKT-R.

Therefore, the present study investigated whether there are patient classes based on psychiatric diagnoses and previously committed offenses, in a nationwide sample of forensic psychiatric patients residing in Dutch high-security forensic psychiatric institutions. In addition, it was also investigated whether risk and protective factors could be detected by the HKT-R that are class-specific and thus significantly more prevalent in one class compared to other classes. A priori, we expected to identify patient classes comparable to the classes found by Van Der Veeken et al. (2017).

## Materials and Methods

### Participants and Procedure

The present study is part of a larger project investigating forensic psychiatric patients who were unconditionally released following mandatory treatment from any of the six Dutch forensic psychiatric centers (FPCs), five forensic psychiatric clinics (FPKs), and one center for transcultural psychiatry (CTP)^1^. A FPC is a maximum secured, closed center where patients stay with a TBS [Terbeschikkingstelling] order. TBS, literally translated as “At the disposal of the Government,” is an entrustment order enshrined in the Dutch Penal Code for mentally disordered offenders who are held not or just partly accountable for their offenses and are considered to stay dangerous for a society without treatment. A FPK and CTP are also closed institutions where, among patients with a different legal title, patients stay with a TBS order. The security level is high, but not as high as in the FPC. All these facilities offer intensive treatment aimed at reducing the risk of reoffending. The data were collected in two sequential studies. In the first study all patients were released between 2004 and 2008; in the second, between 2009 and 2014.

All data within both studies were collected from the electronic patient files containing detailed descriptions of the patient's background and criminal history, diagnoses according to DSM-IV-TR (American Psychiatric Association, 2000), treatment plans, leave requests, and prolongation advice. The data collection was performed by trained coders (Spreen et al., 2014) retrospectively for each patient. For the purpose of the present study, we used measurements concerning the admission to the FPCs. All data were anonymized and could not be linked back to individual patients. The study was approved by the Scientific Research Committee of the FPC Kijvelanden, the Dutch Ministry of Security and Justice, the directors of the FPCs involved in this study and the Ethical Review Board of Tilburg University.

### Measures

#### Criminal History

Information regarding previous crimes for which the patient received a sentence (including the index offense), was derived from the electronic patient files and broken down into 12 categories based on Brand's (2005) so-called BOOG categorization. To facilitate the interpretation of the results, we reduced the number of categories to seven taking into account the severity of offenses: (1) non-violent offenses (*traffic violations and civil disorder, drug-related offenses, destruction of property, and fiscal capital and profit offenses*), (2) light/medium violent offenses (*mild to moderate violence and possession of arms, power by force*), (3) severe violent offenses, (4) sexual offenses against adults, (5) sexual offenses against minors, (6) arson with common danger to persons, and (7) homicide/murder (*manslaughter and premeditated murder*). The number of offenses per variable ranged from 0 to 178 offenses within a patient (Brand, 2005). However, to make the analysis more manageable, we adjusted the scores on these seven variables so that they could range from 0 to 10 offenses, whereas a score of 10 refers to 10 offenses or more. Although the specificity and heterogeneity of the offenses were somewhat lost, we remained with seven types of offenses in our analyses, which is still quite detailed. More detail on the BOOG categories can be found in Supplementary Table 1 in the Supplementary Material.

#### Psychopathology

Psychopathology was based on the presence of the DSM-IV-TR Axis I and II disorders (American Psychiatric Association, 2000) and was determined by a psychiatrist in consultation with a clinical psychologist considering all patients' information that was available at the time of admission to the FPCs. Axis I classifications include all clinical disorders except PDs and ID, which are classified on Axis II. In the present study, Axis I classifications were divided into six categories: no diagnosis on Axis I, mood disorder, developmental disorder, psychotic disorder, SUD, and other disorders. We created dummy variables to allow comorbidity among diagnoses on Axis I. Similarly, seven categories were created for Axis II classifications: no diagnosis on Axis II, cluster A PD, cluster B PD, cluster C PD, PD NOS, multiple PDs, and ID (i.e., IQ ≤ 80).

#### Risk and Protective Factors

Risk and protective factors were assessed with the HKT-R (Spreen et al., 2014). It is a well-validated and most commonly used Dutch risk assessment instrument for assessing 12 Historical, 14 Clinical, and seven Future risk and protective factors for violent reoffending in forensic psychiatric patients. The HKT-R is the revised version of the HKT-30 (Comité Instrumentarium Forensische Psychiatrie, 2000), validated on a Dutch representative group of forensic psychiatric patients. The HKT-R represents an extension of the HCR-20 (Webster et al., 1997), which is the most widely used risk assessment instrument in the world to assess violent risk. In many countries, as well as in the Netherlands, a risk assessment must be performed at least once a year to estimate the future risk of recidivism and change in risk level compared to the previous 12 months of stay in the institution (Bogaerts et al., 2020). In this study, only the 14 Clinical items were used because they are changeable and potentially amenable to treatment. In contrast, historical factors are irreversible and static, while future factors are solely related to the post-release situation. The original clinical scale was divided into seven risk and seven protective factors as was done in the study by Bogaerts et al. (2020).

The risk factors included: psychotic symptoms, addiction, impulsivity, antisocial behavior, hostility, violation of terms, and influence by risky network members. These risk factors were rated on a five-point Likert scale ranging from 0 = *no risk* to 4 = *high risk*. The protective factors included: problem insight, social skills, self-reliance, treatment compliance, taking responsibility for the index offense, coping skills and labor skills. Protective factors were coded reversely, such that 0 = *no protection* and 4 = *high protection*. A comprehensive description of the HKT-R indicators can be found in Supplementary Table 2 in the Supplementary Material. Internal consistency for the Clinical domain proved to be good in the previous research (α = 0.80; Bogaerts et al., 2020) as well as in the current study with Cronbach's alpha coefficient of α = 0.79 (95% CI: 0.76–0.81) and McDonald's Omega coefficient of ω = 0.80 (95% CI: 0.75–0.82).

### Statistical Analysis

First, we computed descriptive statistics of demographic and questionnaire data as well as correlations for all study variables using SPSS version 25 (IBM Corp., Armonk, NY, USA). Subsequently, a three-step LCA was performed in the Latent GOLD version 5.1 (Vermunt and Magidson, 2016), to identify the clinical patient classes and to investigate differences in the 14 clinical HKT-R factors across potential patient classes. The LCA is a form of finite mixture modeling used to identify the potential latent classes of individuals among the set of indicators (McLachlan and Peel, 2004). In the first step, a latent class model was built taking into account psychopathology and criminal history. A decision about the number of latent classes was based on the Bayesian information criterion (BIC), Akaike information criterion (AIC), and the AIC3, where the lower values indicated the better fit. The BIC is considered a more reliable measure compared to AIC and AIC3, because it penalizes free parameters more strongly than the AIC and AIC3 do (Vermunt and Magidson, 2013). After selecting the best fitting model, we estimated the Bootstrap *p-*value to provide a more precise estimation and improved power, where *p* > 0.05 indicated a good fit. In the next step, cases were assigned to the latent classes. The quality of the determined classification was evaluated by the Entropy *R*^2^ such that values closer to one indicated a better predicting model. The −2 log likelihood (−2*LL*) was used to test whether the chosen model provides a significant improvement relative to a model with fewer classes (Vermunt and Magidson, 2005). Finally, in the third step, we investigated between-class differences in the mean scores of the risk and protective factors with the Wald test at the 5% significance level. In order to reduce the likelihood for a Type I error when computing multiple pairwise comparisons, we adjusted the alpha level using a Bonferroni correction (α/10 = 0.005).

## Results

### Sample Characteristics

The combined study sample consisted of 815 patients of which 347 patients (8.6% female) were unconditionally released between 2004 and 2008, and 468 patients (13.5% female) between 2009 and 2014 from any of the 12 Dutch forensic institutions. Because the number of females (*n* = 93, 11.4%) was too small to investigate the clinical patient classes, the present study involved only males. Of the final sample of 722 male patients, 539 (74.6%) were born in the Netherlands, and 183 (25.4%) abroad. The mean age at admission to the FPCs was 32.28 years (*SD* = 9.36, range = 17–79), and on average, patients stayed in the FPCs for 8.25 years (*SD* = 3.45, range = 1–26). The index offenses included manslaughter (*n* = 244, 33.8%), moderate violence (*n* = 216, 29.1%), robbery (*n* = 170, 23.5%), severe violence (*n* = 113, 15.7%), murder (*n* = 111, 15.4%), sexual violence against adults (*n* = 100, 13.9%), arson (*n* = 88, 12.2%), and sexual violence against minors (*n* = 64, 8.9%). Patients could be convicted of multiple index offenses at the same time. The other sample characteristics are presented in Table 1, while descriptive statistics and correlations for clinical risk and protective factors of the HKT-R are displayed in Table 2.

**Table 1:** Sample characteristics.

**Variable**	***M* (*SD*)/*N* (%)**
Age at admission (in years)	32.28 (9.36)
Age at discharge (in years)	40.95 (9.50)
Length of stay (in years)	8.25 (3.45)
**Birthland**	
The Netherlands	539 (74.6%)
Suriname	49 (6.8%)
Curacao	30 (4.2%)
Morocco	26 (3.6%)
Turkey	15 (2.1%)
Elsewhere	63 (8.7%)
**Axis I diagnosis**	
No Axis I diagnosis	218 (30.2%)
Developmental disorders	57 (7.9%)
Substance use disorders	310 (42.9%)
Mood disorder	55 (7.6%)
Schizophrenia and other psychotic disorders	178 (24.7%)
Other Axis I diagnoses	95 (13.2%)
**Axis II diagnosis**	
No Axis II diagnosis	
Cluster A PDs	25 (3.5%)
Cluster B PDs	200 (27.7%)
Cluster C PDs	22 (3.0%)
PD not otherwise specified	305 (42.2%)
Multiple PDs	18 (2.5%)
Intellectual disability	102 (14.4%)
**Index offenses**	
Manslaughter	244 (33.8%)
Moderate violence	216 (29.1%)
Robbery	170 (23.5%)
Severe violence	113 (15.7%)
Murder	111 (15.4%)
Sexual violence against adults	100 (13.9%)
Arson	88 (12.2%)
Sexual violence against minors	64 (8.9%)

*PD, Personality disorders*.

**Table 2:** Descriptive statistics and correlations for clinical risk and protective factors.

**Variable**	***n***	***M***	***SD***	**1**	**2**	**3**	**4**	**5**	**6**	**7**	**8**	**9**	**10**	**11**	**12**	**13**	**14**
1. Psychotic symptoms	684	0.43	0.88	–													
2. Addiction	680	0.48	1.02	−0.06	–												
3. Impulsivity	654	1.81	1.34	0.15^**^	0.23^**^	–											
4. Antisocial behavior	654	1.35	1.28	0.17^**^	0.18^**^	0.50^**^	–										
5. Hostility	646	1.33	1.12	0.27^**^	0.17^**^	0.45^**^	0.43^**^	–									
6. Violation of terms	681	1.16	1.43	0.28^**^	0.29^**^	0.42^**^	0.46^**^	0.48^**^	–								
7. Influence by risky network members	658	1.14	1.36	0.13^**^	0.03	0.02	0.14^**^	0.07	0.14^**^	–							
8. Problem insight	656	1.24	1.00	−0.23^**^	0.01	−0.08^*^	−0.21^**^	−0.19^**^	−0.24^**^	−0.23^**^	–						
9. Social skills	664	2.02	0.94	−0.11^**^	−0.06	−0.30^**^	−0.41^**^	−0.33^**^	−0.26^**^	−0.12^**^	0.21^**^	–					
10. Self-reliance	657	3.38	0.99	−0.32^**^	0.01	−0.13^**^	−0.09^*^	−0.11^**^	−0.13^**^	−0.14^**^	0.20^**^	0.20^**^	–				
11. Treatment compliance	681	2.42	1.24	−0.21^**^	−0.17^**^	−0.27^**^	−0.39^**^	−0.38^**^	−0.41^**^	−0.22^**^	0.46^**^	0.26^**^	0.23^**^	–			
12. Taking Responsibility for index offense	591	1.91	1.28	−0.10^*^	0.02	−0.09^*^	−0.21^**^	−0.15^**^	−0.13^**^	−0.12^**^	0.45^**^	0.13^**^	0.06	0.37^**^	–		
13. Coping skills	656	1.37	0.91	−0.19^**^	−0.14^**^	−0.46^**^	−0.45^**^	−0.39^**^	−0.38^**^	−0.12^**^	0.23^**^	0.44^**^	0.12^**^	0.39^**^	0.18^**^	–	
14. Labor skills	623	3.08	1.24	−0.19^**^	−0.12^**^	−0.24^**^	−0.33^**^	−0.21^**^	−0.25^**^	−0.11^**^	0.20^**^	0.27^**^	0.32^**^	0.41^**^	0.16^**^	0.30^**^	–

* *p < 0.05;*

** *p < 0.005; n, number of participants*.

### Model Estimation

First, to identify the number of classes that provided the best fit to the data, a series of models was tested with each subsequent model evaluating an additional class. In total, six models were estimated (Table 3). Based on the lowest BIC value, a five-class model was selected as most informative: first class (*n* = 217, 30.1%), second class (*n* = 188, 26.1%), third class (*n* = 134, 18.5%), fourth class (*n* = 124, 17.1%), and fifth class (*n* = 59, 8.2%). Lastly, the five-class solution had adequate classification quality, given the estimated proportional classification errors and the Entropy *R*^2^ value.

**Table 3:** Nested model comparisons for latent classes.

**Number of classes**	**BIC (L^**2**^)**	**AIC (L^**2**^)**	**AIC3 (L^**2**^)**	**N*par***	**L^**2**^**	***df***	***p*^a^**	**Class error**	**Entropy of R**
1	5,094.1274	8,004.0649	7,366.0649	69	9,280.0649	638	0.09	0.0000	1.0000
2	4,548.6465	7,362.8024	6,745.8024	90	8,596.8024	617	0.10	0.0001	0.9984
3	4,286.2130	7,004.5872	6,408.5872	111	8,196.5872	596	0.08	0.0005	0.9968
4	4,075.3301	6,697.9228	6,122.9228	132	7,847.9228	575	0.08	0.0030	0.9934
**5**	**4,058.1156**	**6,584.9266**	**6,030.9266**	**153**	**7,692.9266**	**554**	**0.06**	**0.0130**	**0.9687**
6	4,079.7177	6,510.7471	5,977.7471	174	7,576.7471	533	0.09	0.0688	0.9056

*The chosen model is presented in bold. Npar, number of parameters; BIC, Bayesian information criterion; AIC, Akaike information criterion*.

a *p after bootstrapping, indicating model fit when non-significant (≥0.05)*.

### Characteristics of Classes

**Figure 1:**
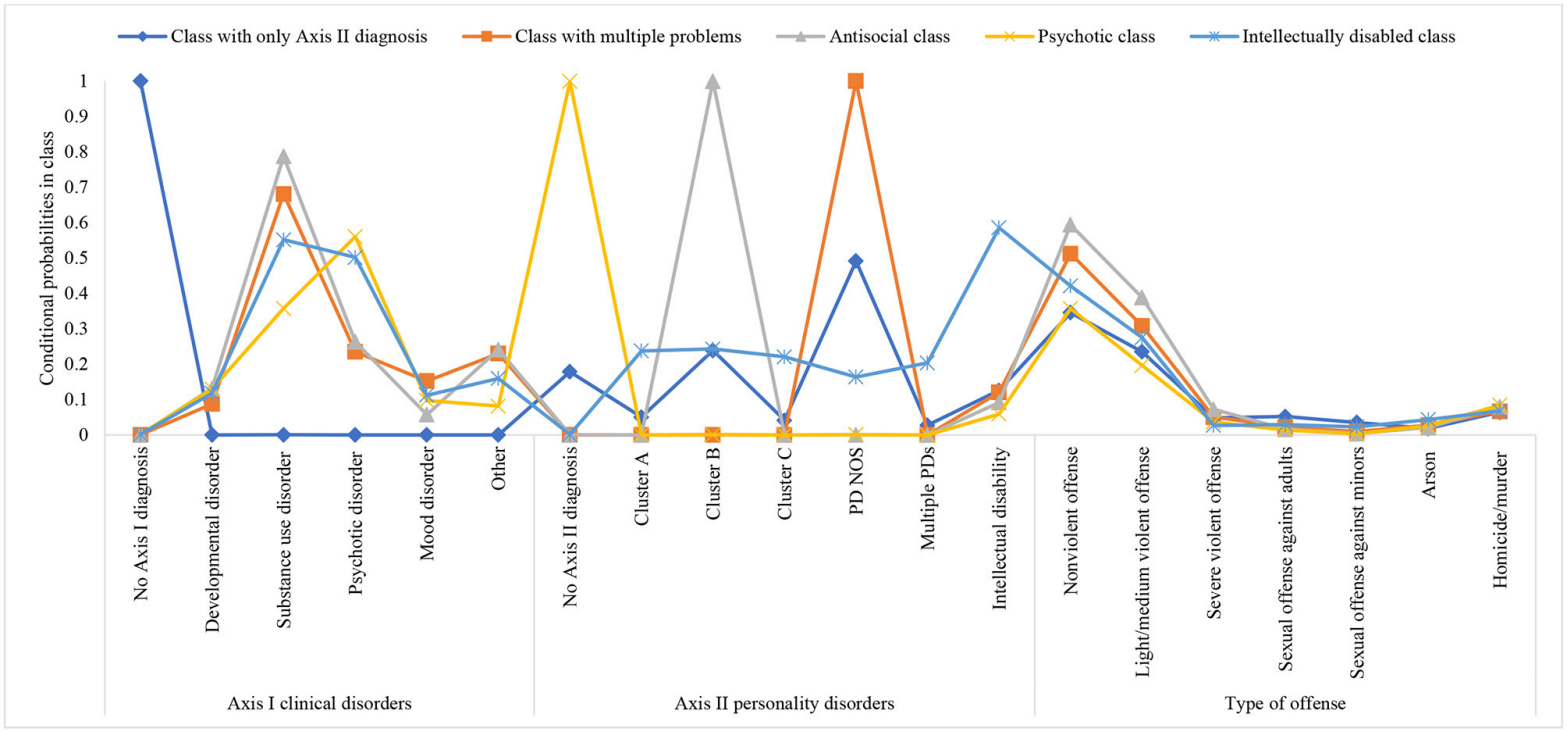
Graphical representation of conditional probabilities by class. PD, personality disorder; NOS, not otherwise specified.

In the second step, cases were assigned to the latent classes. The resulting classes are displayed in Table 4 and Figure 1. The largest class (first class) was labeled the *class with only Axis II diagnosis*. Compared to the other classes, this class was characterized by patients with no Axis I diagnosis. On Axis II, patients were likely to have a diagnosis of PD NOS. The prevalence of criminal offenses in this class was comparable with the prevalence of those in the other four classes, with the most common offenses in category 1 (non-violent) and category 2 (light/medium violent offenses). Finally, this class was characterized by a history of sexual offenses against adults as well as minors.

**Table 4:** Class-specific probabilities/means of the psychopathology and type of offense.

	**Class with only axis II diagnosis (*n* = 217)**	**Class with multiple problems (*n* = 188)**	**Antisocial class (*n* = 134)**	**Psychotic class (*n = 124*)**	**Intellectually disabled class (*n* = 59)**	**Wald test for paired comparisons**
	***P (SE)***	***P (SE)***	***P (SE)***	***P (SE)***	***P (SE)***	
**NOMINAL INDICATORS**						
**Axis I diagnosis**						
No diagnosis	0.99 (0.00)	0.00 (0.00)	0.00 (0.00)	0.00 (0.01)	0.00 (0.00)	1 > 2, 3, 4, 5
Developmental disorder	0.00 (0.00)	0.09 (0.02)	0.13 (0.03)	0.13 (0.03)	0.12 (0.05)	*ns*
Substance use disorder	0.00 (0.00)	0.68 (0.03)	0.79 (0.04)	0.36 (0.04)	0.55 (0.07)	2 > 4; 3 > 4, 5
Psychotic disorder	0.00 (0.00)	0.24 (0.03)	0.26 (0.04)	0.56 (0.04)	0.50 (0.07)	2, 3 <4, 5
Mood disorder	0.00 (0.00)	0.15 (0.03)	0.06 (0.02)	0.10 (0.03)	0.11 (0.04)	*ns*
Other disorders	0.00 (0.00)	0.23 (0.03)	0.24 (0.04)	0.08 (0.02)	0.16 (0.05)	4 <2, 3
**Axis II diagnosis**						
No diagnosis	0.18 (0.03)	0.00 (0.00)	0.00 (0.00)	0.99 (0.00)	0.00 (0.01)	4 > 1, 2
Cluster A PD	0.05 (0.01)	0.00 (0.00)	0.00 (0.00)	0.00 (0.00)	0.24 (0.06)	5 > 1
Cluster B PD	0.24 (0.03)	0.00 (0.00)	0.99 (0.00)	0.00 (0.00)	0.24 (0.07)	3 > 1, 2, 4, 5
Cluster C PD	0.04 (0.01)	0.00 (0.00)	0.00 (0.00)	0.00 (0.00)	0.22 (0.06)	5 > 1
PD NOS	0.49 (0.03)	0.99 (0.00)	0.00 (0.00)	0.00 (0.00)	0.16 (0.06)	2 > 3, 4, 5; 1 > 5
Multiple PDs	0.03 (0.01)	0.00 (0.00)	0.00 (0.00)	0.00 (0.00)	0.20 (0.05)	5 > 1
Intellectual disability	0.13 (0.02)	0.12 (0.03)	0.09 (0.03)	0.06 (0.02)	0.59 (0.07)	5 > 1, 2, 3, 4,
**ORDINAL INDICATORS**						
**Type of offense**						
Non-violent offense	3.45 (0.25)	5.12 (0.30)	5.94 (0.35)	3.57 (0.34)	4.21 (0.55)	1, 2 <3; 2, 3 > 4
Light/Medium violent offense	2.35 (0.19)	3.08 (0.24)	3.88 (0.31)	1.96 (0.23)	2.75 (0.43)	1 <3; 4 <2, 3
Severe violent offense	0.48 (0.06)	0.51 (0.07)	0.72 (0.11)	0.37 (0.07)	0.27 (0.09)	*ns*
Sexual offense against adults	0.52 (0.15)	0.22 (0.06)	0.16 (0.05)	0.14 (0.05)	0.29 (0.14)	*ns*
Sexual offense against minors	0.35 (0.10)	0.10 (0.04)	0.04 (0.02)	0.03 (0.02)	0.23 (0.16)	*ns*
Arson	0.18 (0.04)	0.28 (0.06)	0.21 (0.06)	0.26 (0.07)	0.44 (0.16)	*ns*
Homicide/murder	0.64 (0.05)	0.67 (0.06)	0.79 (0.08)	0.85 (0.09)	0.68 (0.12)	*ns*

*n, number of participants; P, probability; SE, standard error; M, Mean; PD, personality disorder; NOS, not otherwise specified; ns, non-significant. Significant differences between classes according to the Wald test for paired comparisons are Bonferroni corrected at p < 0.005*.

The second class was labeled the *class with multiple problems*. Patients in this class were highly likely to have SUD. In addition, this class was characterized by mood disorders and “other” Axis I diagnoses. This class also had the greatest probability of having PD NOS compared to all the other classes. Lastly, patients in this class were likely to have a history of non-violent, light/medium, and severe violent offenses.

The *antisocial class* was the third class (in line with Van Der Veeken et al., 2017). On Axis I, this class had the highest probability of having a diagnosis of SUD. In addition, patients in this class were also likely to have “other” Axis I diagnoses. Moreover, this class had the highest probability of having a cluster B PD diagnosis compared to the other classes. Finally, patients in this class were likely to have committed non-violent, light/medium, and severe violent offenses as well as homicide/murder.

The fourth class was the *psychotic class*. Patients in this class had the highest probability of having a psychotic disorder and no Axis II diagnosis compared to the other classes. As in the other classes, the predominant criminal offenses were non-violent offense and light/medium violence. However, patients in this class had the greatest probability of having committed homicide/murder.

Finally, the smallest class was the *intellectually disabled class*. On Axis I, patients were likely to be diagnosed with SUD and psychotic disorder. When it comes to Axis II, patients in this class were most likely to be diagnosed with ID, compared to patients in the other classes. They were also most likely to have cluster A PD and/or cluster C PD. In addition, patients in this class had a greater probability of committing sexual offenses and the highest probability of committing arson relative to the patients in the other classes. More details about pairwise comparisons on internalizing indices (i.e., Axis I and II diagnoses, and criminal history) can be found in Table 4, while demographic and clinical characteristics per class are displayed in Table 5.

**Table 5:** Demographic and clinical characteristics per class.

	**Class with only Axis II diagnosis (*n* = 217)**	**Class with multiple problems (*n* = 188)**	**Antisocial class (*n* = 134)**	**Psychotic class (*n* = 124)**	**Intellectually disabled class (*n* = 59)**	**Test statistic**
Mean age at admission in years (*SE*)	30.67 (9.17)	34.12 (9.48)	31.47 (8.69)	32.66 (10.03)	33.55 (8.56)	*F*_(4,717)_ = 4.070^*^
Mean age at discharge in years (*SE*)	40.01 (9.59)	42.67 (9.61)	39.77 (9.01)	41.03 (9.88)	41.59 (8.38)	*F*_(4,712)_ = 2.714^*^
**Nationality (%)**						*χ^2^* = 9.777^*^
Dutch	29.0%	25.0%	17.6%	14.8%	6.1%	
Other	1.2%	1.7%	1.7%	2.1%	0.8%	
Violent recidivists within 2 years after release (%)	3.1%	4.2%	5.4%	2.5%	1.3%	*χ^2^* = 18.663^*^
**Axis I diagnosis (%)**						
Developmental disorders (7.9%)	0.0%	2.4%	2.5%	2.2%	0.8%	*χ^2^* = 29.395^**^
Mood disorder (7.6%)	0.0%	4.0%	1.1%	1.7%	0.8%	*χ^2^* = 36.116^**^
Substance use disorders (42.9%)	0.0%	18.0%	14.9%	6.1%	3.9%	*χ^2^* = 286.730^**^
Schizophrenia and other psychotic disorders (24.7%)	0.0%	6.3%	5.3%%	9.6%	3.5%	*χ^2^* = 154.681^**^
Other axis I diagnoses (13.2%)	0.0%	6.1%	4.6%	1.4%	1.1%	*χ^2^* = 65.732^**^
**Axis II diagnosis (%)**						
Cluster A PDs (3.5%)	1.6%	0.0%	0.0%	0.0%	1.9%	*χ^2^* = 107.798^**^
Cluster B PDs (27.7%)	7.2%	0.0%	19.3%	0.0%	1.2%	*χ^2^* = 487.441^**^
Cluster C PDs (3.0%)	1.2%	0.0%	0.0%	0.0%	1.8%	*χ^2^* = 104.297^**^
PD not otherwise specified (42.2%)	14.8%	26.6%	0.0%	0.0%	0.8%	*χ^2^* = 477.059^**^
Multiple PDs (2.5%)	0.8%	0.0%	0.0%	0.0%	1.7%	*χ^2^* = 106.807^**^
Intellectual disability (14.4%)	3.7%	3.3%	1.9%	1.1%	4.4%	*χ^2^* = 97.114^**^

*n, number of participants; PD, personality disorder*.

* *p < 0.05;*

** *p < 0.001*.

### Class Specific Risk and Protective Factors

Finally, in the third step, we investigated between-class differences in the mean scores of the risk and protective factors assessed with both scale and item scores.

#### Class Comparisons at the Scale Level

As shown in Table 6, there were significant class differences in the mean scores of the risk and protective factors at the scale level, respectively (Wald = 11.49, *p* = 0.02; Wald = 14.38, *p* < 0.001). The antisocial class had the highest mean on *risk factors* compared to the other classes. It was followed in descending order by the psychotic class, the class with multiple problems, the intellectually disabled class and the class with only Axis II diagnosis.

**Table 6:** Class-specific means of risk and protective factors at scale level.

	**Class with only Axis II diagnosis**	**Class with multiple problems**	**Antisocial class**	**Psychotic class**	**Intellectually disabled class**	**Wald**	***p***	**Wald test for paired comparisons**
***M (SD)***								
Risk factors	6.92 (0.38)	7.79 (0.37)	8.96 (0.48)	8.13 (0.52)	7.58 (0.83)	11.49	0.02	1 < 3
Protective factors	15.11 (0.37)	16.14 (0.37)	16.09 (0.44)	14.34 (0.46)	13.93 (0.90)	14.38	<0.001	2 > 4

*PD, Personality disorder. Significant differences between classes according to the Wald test for paired comparisons are at the level p < 0.005*.

Moreover, the class with multiple problems had the highest mean on *protective factors* in comparison to the other classes. It was followed in descending order by the antisocial class, the class with only Axis II diagnosis, the psychotic class, and the intellectually disabled class. The pairwise comparisons on risk and protective factors at the scale level are displayed in Table 6.

#### Class Comparisons at the Item Level

Considering risk factors at the item level, the Wald statistic (Table 7) showed that there were significant between-class differences in the mean scores of psychotic symptoms (Wald = 53.66; *p* < 0.001), addiction (Wald = 14.92; *p* < 0.001), impulsivity (Wald = 13.00, *p* = 0.01), antisocial behavior (Wald = 10.86, *p* = 0.03), and influence by risky network members (Wald = 11.06, *p* = 0.03).

**Table 7:** Class-specific means of risk and protective factors at item level.

**Mean (*SD*)**	**Class with only Axis II diagnosis**	**Class with multiple problems**	**Antisocial class**	**Psychotic class**	**Intellectually disabled class**	**Wald**	***p***	**Wald test for paired comparisons**
**RISK FACTORS**								
Psychotic symptoms	0.12 (0.03)	0.31 (0.05)	0.37 (0.08)	1.09 (0.11)	0.61 (0.14)	53.66	<0.001	1 <2, 3, 4, 5; 4 > 2, 3
Addiction	0.45 (0.07)	0.56 (0.08)	0.72 (0.11)	0.29 (0.07)	0.17 (0.10)	14.92	<0.001	3 > 4
Impulsivity	1.70 (0.10)	1.85 (0.10)	2.17 (0.12)	1.64 (0.13)	1.58 (0.20)	13.00	0.01	3 > 1, 4
Antisocial behavior	1.33 (0.09)	1.42 (0.10)	1.62 (0.11)	1.16 (0.12)	1.02 (0.18)	10.86	0.03	*ns*
Hostility	1.25 (0.08)	1.43 (0.09)	1.42 (0.10)	1.21 (0.10)	1.42 (0.17)	4.38	0.36	*ns*
Violation of terms	1.08 (0.10)	1.07 (0.11)	1.46 (0.13)	1.16 (0.14)	0.95 (0.19)	7.86	0.10	*ns*
Risky network members	0.93 (0.09)	1.09 (0.10)	1.21 (0.13)	1.44 (0.13)	1.19 (0.21)	11.06	0.03	4 > 1
**PROTECTIVE FACTORS**								
Problem insight	1.23 (0.07)	1.46 (0.08)	1.27 (0.09)	1.06 (0.10)	0.93 (0.14)	14.52	<0.001	2 > 4, 5
Social skills	1.93 (0.07)	2.12 (0.07)	2.08 (0.09)	2.05 (0.08)	1.83 (0.13)	6.30	0.18	*ns*
Self-reliance	3.56 (0.06)	3.48 (0.07)	3.54 (0.08)	2.88 (0.11)	3.15 (0.17)	39.44	<0.001	1 > 4, 5; 4 <2, 3
Treatment compliance	2.42 (0.09)	2.59 (0.09)	2.34 (0.11)	2.25 (0.12)	2.40 (0.18)	6.16	0.19	*ns*
Taking Responsibility for index offense	1.85 (0.09)	1.99 (0.10)	1.93 (0.12)	1.97 (0.13)	1.66 (0.21)	2.42	0.66	*ns*
Coping skills	1.25 (0.06)	1.43 (0.06)	1.34 (0.09)	1.45 (0.09)	1.49 (0.11)	5.80	0.21	*ns*
Labor skills	3.02 (0.09)	3.21 (0.09)	3.20 (0.10)	2.87 (0.13)	3.02 (0.23)	6.41	0.17	*ns*

*PD, Personality disorder; ns, non-significant. Significant differences between classes according to the Wald test for paired comparisons are Bonferroni corrected at p < 0.005*.

The antisocial class had the highest levels of *addiction, impulsivity*, and *antisocial behavior*. In decreasing order, it was followed by the class with multiple problems, the class with only Axis II diagnosis, the psychotic class and the intellectually disabled class. Although the psychotic class scored somewhat lower on addiction, impulsivity and antisocial behavior, it displayed the highest levels of *psychotic symptoms* and *risky network members* compared to all other classes. Similarly, the intellectually disabled class also scored fairly high on psychotic symptoms, followed in decreasing order by the antisocial class, the class with multiple problems and the class with only Axis II diagnosis. Moreover, the latter had also the lowest score on risky network members, followed in ascending order by the class with multiple problems, the intellectually disabled class and the antisocial class.

Moreover, Wald statistics on item-level protective factors showed that there were significant between-class differences in the mean scores of problem insight (Wald = 14.52, *p* < 0.001) and self-reliance (Wald = 39.44, *p* < 0.001), respectively. The class with multiple problems scored the highest on *problem insight* compared to the other classes. Likewise, the class with only Axis II diagnosis and the class with multiple problems scored also very high on problem insight, while the psychotic class and the intellectually disabled class were characterized by somewhat lower scores on this factor. Finally, the psychotic class scored the lowest on *self-reliance*. In comparison to the psychotic class, the intellectually disabled class scored somewhat higher on self-reliance, whereas the other three classes scored considerably higher on this factor. The pairwise comparisons on risk and protective factors at the item level are displayed in Table 7.

## Discussion

In this study, we performed LCA in a nationwide heterogeneous group of Dutch male forensic psychiatric patients in order to classify more homogeneous classes that might prove useful for future, class tailored or even personalized, interventions. Subsequently, we investigated which specific risk and protective factors were characteristic for each class. Five distinctive patient classes emerged: *class with only Axis II diagnosis, class with multiple problems, antisocial class, psychotic class*, and *intellectually disabled class*. Significant differences were found for risk and protective factors at both scale level and item level across classes. Classes differed significantly in the mean scores of psychotic symptoms, addiction, impulsivity, antisocial behavior, and influence by risky network members as well as in the mean scores of problem insight and self-reliance.

Overall, the results largely support the previously established patient classes found by Van Der Veeken et al. (2017), and show similarities with other studies (Bogaerts and Spreen, 2011; Van Nieuwenhuizen et al., 2011; Schmitter et al., 2021). However, notable differences were also evident. First, we have identified five classes in the current study, while Van Der Veeken et al. (2017) found four patient classes in their research. This distinction might be attributed to a different and smaller sample in the latter study that included only two FPCs. In comparison with Van Der Veeken et al. (2017), the most notable finding of the current study is an intellectually disabled class. This could be due to the fact that they only included PDs in their study and did not include ID as a diagnosis. Individuals with ID have rarely been studied in forensic settings, but the existing evidence supports the assumption that forensic patients diagnosed with ID do indeed exhibit distinctive characteristics and behaviors compared to patients without this diagnosis (Ray et al., 2019). For example, it has been found that, compared to non-ID patients, patients with ID are more likely to commit sexual offenses and arson (Männynsalo et al., 2009; Lunsky et al., 2011; Ray et al., 2019), which was documented in our study as well. This higher rate of sexual offenses among ID patients has been attributed to their lower social awareness and behavioral self-control (Männynsalo et al., 2009). Besides, it is well-recognized that ID patients have interpersonal and learning difficulties, meaning that they may require a different approach and more intensive support than patients without ID. Therefore, interventions must be tailored to the criminogenic needs, learning style, motivation, and abilities of these offenders, as outlined by the responsivity principle of the RNR model (Andrews et al., 2011). For example, in our study, a *post-hoc* analysis revealed that patients in the intellectually disabled class have more difficulties recognizing their risky behaviors than patients in the class with multiple problems. In addition, patients with ID struggled more in performing daily tasks independently compared to patients in the other classes, with the exception of patients in the psychotic class, who struggled most with performing these activities independently. Thus, the treatment of patients with ID should focus more on deficiencies in the domain of self-reliance as well as on gaining insight into what drives their behavior and which risk situations can lead to reoffending (Spreen et al., 2014). Finally, the present study showed that the intellectually disabled class was characterized by lower levels of both risk and protective factors at the scale level compared to the other classes. This signifies that targeting protective factors during treatment might be beneficial for this class of ID patients.

Furthermore, consistent with previous studies (Van Nieuwenhuizen et al., 2011; Van Der Veeken et al., 2017), we also identified the *antisocial class*. However, opposed to the study by Van Der Veeken et al. (2017), the antisocial class in our study was characterized by higher levels of risk factors at the scale level, compared to the other classes. Likewise, at the item level, this class displayed the highest levels of antisocial behavior, impulsivity and addiction. Many previous studies have consistently related substance use, antisocial behavior, and greater levels of impulsivity to cluster B PDs, which is the main characteristic of the antisocial class identified in this study (e.g., Perry and Körner, 2011; Goretti et al., 2017; Schmitter et al., 2021). It could be that poor impulse control and addiction problems put patients in the antisocial class at risk for more serious violent offenses than patients in the other classes. To illustrate, impaired emotion regulation capacity and maladaptive emotional response to both stressful and social situations of cluster B patients may enforce the impulsive symptoms to emerge. This could further lead to alcohol and drug addiction, and the progression of dramatic overly emotional or unpredictable thinking, feeling, or behavior, and consequently to the development of aggressive behavior and violence (Douzenis et al., 2012; Jankovic et al., 2021). Besides, poor self-regulation and higher impulsivity are thought to be the most significant explanatory factors for criminal behaviors according to the general theory of crime (Gottfredson and Hirschi, 1990). In addition, addiction, impulsivity, and antisocial behavior are, according to the RNR model (Andrews and Bonta, 2006, 2010), the most important factors for predicting violent reoffending. Indeed, as shown in Table 5, the antisocial class has the highest recidivism rate compared to the other classes. Hence, additional research is needed to investigate if these particular risk factors, that is, impulsivity, addiction, and antisocial behavior, led to reoffending in the antisocial class. Moreover, the pairwise comparison test revealed that patients in the antisocial class struggled less with accomplishing daily activities compared to the psychotic class and the intellectually disabled class. This finding corresponds with previous research showing that personal care was the least disrupted and the most satisfying self-care activity in cluster B patients (Larivière et al., 2010). Last but not least, the antisocial class scored higher on protective factors at the scale level, compared to all other classes, except for the class with multiple problems, which scored the highest. This signifies that offenders in this class of antisocial patients might benefit the most if treatment focuses primarily on targeting risk factors, particularly addiction, antisocial behavior, and impulsivity.

Moreover, the present study also supports the previously established existence of the *psychotic class* (Bogaerts and Spreen, 2011; Van Nieuwenhuizen et al., 2011; Van Der Veeken et al., 2017). This class is consistent with the *typical psychotic patient* found by Van Nieuwenhuizen et al. (2011) and resembled the *psychotic cluster* found by Bogaerts and Spreen (2011), and the *psychotic first offender class* found by Van Der Veeken et al. (2017). Yet, there is a clear difference between the psychotic first offender class and the psychotic class identified in our study considering Axis II diagnoses. That is, patients in the psychotic class from our study did not have a comorbid Axis II diagnosis, whereas patients in the psychotic first offender class of Van Der Veeken et al. (2017), could, however, have a comorbid Axis II diagnosis of cluster A or C PD, or PD NOS. However, it could be noticed that the latter has some overlap with our class of ID patients. Particularly, ID patients were also likely to be diagnosed with psychotic disorder, and/or cluster A PD, and/or cluster C PD. Thus, this highlights the importance of taking ID into account when examining patient classes. In addition, some studies also indicated that it is important to consider the disease onset of psychotic patients in relation to criminal behavior (Hodgins et al., 2013; Van Dongen et al., 2015). For example, these studies found even more specific subgroups of psychotic offenders depending on whether they begin to engage in criminal behavior before the onset of psychosis (early starters), after psychosis onset but at age 34 years or younger (late starters), and after psychosis onset at age 35 years or older (late first offenders). These subgroups also differed in symptomatology and substance abuse. Moreover, we found that the psychotic class scored significantly higher on risk factors and significantly lower on protective factors compared to the other classes. A *post-hoc* analysis further showed that this class displayed significantly higher levels of psychotic symptoms compared to the other classes. The higher levels of psychotic symptoms could be attributed to the underlying psychotic disorder, which is the main characteristic of this class. Patients in this class were also more likely to have risky network members than the class with only Axis II diagnosis and the class with multiple problems as well as to be less self-reliant than the class with multiple problems and the antisocial class. Individuals with schizophrenia or other psychotic disorders are highly likely to be marginalized by society but also by themselves. In addition, due to their condition, they can experience severe and long-term consequences such as unemployment, addiction, poverty, and homelessness (Evensen et al., 2016; Habánik, 2018; Ayano et al., 2019), which could explain why patients in the psychotic class are more likely to have risky network members. Moreover, our findings are in line with the study of Bogaerts et al. (2020), which also found that psychotic symptoms can indeed diminish the patient's ability to complete essential daily tasks independently. In sum, our findings suggest that reducing psychotic symptoms, creating a more prosocial environment, and increasing self-reliance might be the crucial treatment targets of patients who belong to the psychotic class.

Furthermore, we identified the *class with only Axis II diagnosis*, which was shown to be similar to the *maladaptive disordered affective class* found by Van Der Veeken et al. (2017) and to the *patient with sexual problems and sexual crimes* found by Van Nieuwenhuizen et al. (2011). However, these classes found in previous studies could have a diagnosis on Axis I, particularly a diagnosis of sexual-/gender identity disorder or a pervasive developmental disorder. In this study, we also found that the class with only Axis II diagnosis had the lowest mean score on risk factors at the scale level compared to the other classes, and a higher mean score on protective factors at the scale level compared to most other classes. Further analysis revealed that this class scored significantly lower on impulsivity than the antisocial class, and significantly lower on psychotic symptoms than the other classes. This finding signifies that impulsivity and psychotic symptoms are more characteristic to patients who committed severe violent and homicide-related offenses, rather than to sexual offenders. Lastly, the class with only Axis II diagnosis scored significantly higher on self-reliance than the psychotic class. This finding indicates that deficits in the realm of self-reliance are more common in patients with a psychotic disorder, than in patients with PDs. In support of this argument, we also found that the antisocial class and the class with multiple problems scored significantly higher on self-reliance than the psychotic class. In sum, self-reliance might serve as a protective factor against reoffending in patients belonging to the class with only Axis II, the antisocial class and the class with multiple problems.

Finally, we identified the *class with multiple problems*, which has the least resemblance to the patient classes identified in previous studies, although there is some similarity. That is, this class resembled the *patient suffering from addiction* class to some extent (Van Nieuwenhuizen et al., 2011), as patients in this class were also characterized by SUD and PD NOS. Considering risk factors at the scale level, this class was somewhere in the middle compared to the other classes, but had the highest mean compared to the other classes when it comes to protective factors at scale level. It could be that this class has more protection against reoffending in comparison to the other classes because patients in this class do not meet the full criteria of any of the officially recognized diagnostic categories of PDs, but only have some features of one or more PDs. It might be that patients diagnosed by one of the officially recognized PDs have a more pronounced rigid and unhealthy pattern of thinking, functioning, and behavior than patients with PD NOS, and thus less protection. The same cannot be said for the psychotic class, which although was less likely to have any of PDs, has less protection compared to most other classes. However, the psychotic class had the highest probability of having schizophrenia or other psychotic disorder which can cause significant cognitive impairments and lower overall quality of life and hence less protection (Alptekin et al., 2005). Furthermore, a *post-hoc* analysis showed that patients in the class with multiple problems are more aware of their risky behaviors in situations that can lead to relapse than patients in the psychotic class and the intellectually disabled class. This means that better problem insight may serve as a protective factor against committing more severe violent offenses in patients belonging to this class. In brief, targeting risk factors might be the most valuable in the treatment of offenders belonging to the class with multiple problems.

### Clinical Implications

In accordance with the principles of personalized treatment, the findings of this study may be relevant to clinical practice as treatment interventions can be better tailored to the specific needs of these five homogeneous classes. For example, we distinguished a group of patients with intellectual deficits that can have a profound negative impact on their intellectual (e.g., problem-solving), practical (e.g., performing work), and social (e.g., making friends) functioning. Although there is no cure for this condition, appropriate interventions could help these patients improve their functioning. It has been suggested that treatment of these patients should primarily focus on improving their strengths (Cobb et al., 2013). This is also supported by our finding showing that ID patients were characterized with less protection (i.e., strengths) compared to the other patients. Therefore, treatment of these patients should indeed focus more on improving their strengths, which can also serve as a buffer against criminal behavior. In addition, we also found that these patients were more likely to have a history of sexual offenses. Hence, due to the manipulative nature of these types of offenders, it has therefore been suggested that group therapy in these patients is more effective than individual therapy. In the FPCs, patients receive different treatment options, such as cognitive behavioral therapy, schema focus therapy, psychomotor therapy, music therapy, psychopharmaceutical therapy, and a combination of therapies (Van Der Veeken et al., 2017). Future studies may want to investigate which of these therapies are most beneficial for each patient class. Last but not least, our findings provide support for both offender rehabilitation models, that is, the RNR model (Andrews and Bonta, 2010) and the GLM (Ward et al., 2007). Therefore, as stated in previous research, the RNR model and GLM should be indeed viewed as complementary rather than opposing. Thus, by promoting the merits of each, treatment effects could be maximized (Bogaerts et al., 2020).

### Limitations and Directions for Future Research

The present study is not without limitations. First, clinical indicators of the HKT-R were coded retrospectively using official patient files. Although these files contain a wide range of relevant information, the assessment of the HKT-R on direct behavioral observations would have, however, provided more accurate data. Second, despite a large nationwide sample of forensic psychiatric patients that was included in this study, the number of females was too small to investigate clinical patient classes. Therefore, our findings can only be generalizable to the population of Dutch male forensic patients. Female offenders are in general underrepresented in forensic research. Thus, it would be of added value to investigate patient classes in female forensic patients. For example, in our sample, we found some notable gender differences in demographics, psychopathology, criminal history, and risk and protective factors (for an overview of gender difference, see Supplementary Table 3 in the Supplementary Material). It could therefore be speculated that different patient classes might appear in a sample of females. Third, the study was also limited by the use of the DSM-IV-TR, which is nowadays obsolete and replaced by the DSM-5 (American Psychiatric Association, 2013). However, at the time when the study was conducted, the DSM-5 still was not available. One of the biggest differences between these two is that the DSM-5 did not utilize a multiaxial system of diagnosis, but rather combined axes I to III into a single axis representing mental and other medical diagnoses. Nonetheless, from our point of view, these differences probably do not negatively impact the generalizability of the study's findings. Another important limitation is that the design of the study was cross-sectional which makes it impossible to draw causal conclusions about the association between clinical HKT-R indicators and class membership. Future studies would benefit from investigating these associations longitudinally as it could provide more insight into class-specific treatment trajectories of risk and protective factors over time. In addition, researchers may also consider investigating which specific risk and protective factors lead to reoffending across these classes in future studies. Last but not least, our study pointed to potentially valuable treatment targets for each class of patients. Hence, future studies may wish to investigate whether incorporating them into treatment would lead to reduced reoffending. Apart from this, it would also be valuable to gain insight into cognitive deficits in these patient classes, as they can have a major impact on the response to therapeutic intervention. Although these deficits are widespread in psychiatric disorders, not all disorders are equally affected. For example, individuals with schizophrenia have significant impairments in a wide range of cognitive domains, including memory, executive functions, and attention, while cluster B patients have fewer impairments, such as poor decision-making skills and low task orientation (Trivedi, 2006).

### Conclusion

To conclude, in the present study, we distinguished five patient classes in a large heterogeneous sample of male forensic psychiatric patients. Most importantly, the current study identified the existence of the intellectually disabled class, which can be of great importance in clinical practice. Four of the five classes were also found in previous studies with smaller sample sizes, although with some differences. In addition, the evidence is presented that stresses the importance of considering both risk and protective factors for the patient classes.

Finally, these findings indicate there are important differences in risk and protective factors between the five identified patient classes. This suggests that personalized treatment based on class membership may be more effective at decreasing the risk of reoffending compared to general, non-individualized treatment.

## Data Availability Statement

The raw data supporting the conclusions of this article will be made available by the authors, without undue reservation.

## Ethics Statement

The studies involving human participants were reviewed and approved by the Scientific Research Committee of the FPC Kijvelanden, the Dutch Ministry of Security and Justice, the directors of the FPCs involved in this study and the Ethical Review Board of Tilburg University, the Netherlands. The participants provided their written informed consent to participate in this study.

## Author Contributions

MJ analyzed the data and wrote the first draft of the manuscript. SB, EM, MS, and PL critically revised the manuscript for important intellectual content. All authors contributed to and have approved the final manuscript.

## Conflict of Interest

The authors declare that the research was conducted in the absence of any commercial or financial relationships that could be construed as a potential conflict of interest.

## Footnotes

^1^We use the abbreviation FPC(s) later on because all patients have in common that they received a TBS order imposed by the judge.
