# Supplementary material for: A Latent Class Analysis of Forensic Psychiatric Patients in Relation to Risk and Protective Factors
Source: Front Psychol. 2021 Jul 20;12:695354. doi: 10.3389/fpsyg.2021.695354 (PMC8329083; doi:10.3389/fpsyg.2021.695354)
Supplement: Supplementary file 1 [file Data_Sheet_1.docx]

Supplementary Material

# Supplementary Tables

**Table S1**

Categorization of Criminal Offences Based on Brand's (2005) Categorization

| Category | Examples of offences |
| --- | --- |
| 1. Traffic violations and civil disorder | Traveling without a valid ticket, driving without a valid license, driving a vehicle without valid insurance, sedition, abuse based on race, religion and other factors, trespassing, and breach of the peace |
| 2. Drug-related offences | Drug possession and drug distribution |
| 3. Destruction of property | Destruction of property |
| 4. Fiscal capital and profit offences | Incorrect tax declarations, stealing, and fraud |
| 5. Mild to moderate violence and possession of arms | Arson, supporting escape, violent abuse, and threatening behavior |
| 6. Power by force | Stealing with violence or threat and extortion with violence or threat |
| 7. Severe violence | Grave bodily harm and premeditated physical violence. |
| 8. Moral offences | Adult rape, intercourse with an unconscious or mentally handicapped individual, and sexual offences with physical harm |
| 9. Moral offences (with minors as victims) | Child rape, child molestation and forceful sexual intercourse of victim with a third party |
| 10. Manslaughter | Manslaughter |
| 11. Arson | Arson within the context of premeditated harm or death of an individual |
| 12. Premeditated murder | Premeditated murder |

*Note*. This division is based on Brand, E.F.J.M. (2005). *Onderzoeksrapport PIJ-Dossiers 2003C*. Den Haag, DJI

**Table S2**

Description of Clinical Risk and Protective Factors (Spreen et al., 2014)

| Risk factors |  |
| --- | --- |
| K01: Psychotic symptoms | The extent to which the patient showed active psychotic symptoms such as delusions (e.g., hearing voices). Particular attention should be given to symptoms or delusions containing aspects of violence, paranoid content and/or with aspects of executing control over fear. |
| K02: Addiction | Includes alcohol, soft drugs (e.g., cannabis), hard drugs (e.g., heroin, cocaine, speed, LSD), and other addictions such as gambling, sex, internet, or medication. Considering the first three categories, assessment focus is on the frequency of the infringing consumption. If the controlled use of substances is part of the treatment, then it is not seen as infringing consumption. |
| K03: Impulsivity | The extent to which the patient behaved in an unpredictable and/or thoughtless way at the expense of himself or others (“first act, then think”). It can be expressed as irascibility (to have a short fuse), incontrollable immediate need-satisfaction desires (impulse-buys), or a chaotic manner of living. |
| K04: Antisocial behavior | The extent to which the patient pursued his own desires and needs without either taking others’ feelings and needs or the circumstances into account and the extent to which this resulted in transgressive behavior towards others. |
| K05: Hostility | The extent to which the patients attributed hostility to others, along with systems and authorities. Hostility can be manifested in the form of violent perceptions, passive aggression, cynicism, and annoyance, and sometimes in the form of severe verbal and physical aggression. |
| K06: Violation of terms and agreements | The extent to which the patient adhered to his terms and agreements of his treatment. This includes the severity as well as the frequency of potential breaches of treatment agreements, the institution’s rules, or furlough conditions. |
| K07: Influence by risky network members | The extent to which the patient allows himself to be influenced by others in a negative way, including fellow patients, family, friends etc. Risky networks can influence the patient to engage in risky situations or risky behavior. |
| Protective factors |  |
| K08: Problem insight | The extent to which the patient was aware of his individual risk factors and signals of risky behaviors in situations that can lead to his relapse, and the extent to which the patient behaves based on his awareness and knowledge. |
| K09: Social skills | The extent to which the patient was able to maintain social contact with his life- and work- environment in a satisfactory manner (e.g., communication skills, the proper use of manners, assertive behavior during contact with others, appropriate personal space during conversations, etc.). |
| K10: Self-reliance | The extent to which the patient was able to complete essential daily tasks independently, such as personal hygiene, dealing with money, patterns of diet, sleep patterns, self-presentation, care for his surroundings, and the ability to call for medical help. |
| K11: Treatment compliance | The extent to which the patient was involved in his treatment progression. It includes patient’s cooperation, participation during treatment components, the acceptance of rules, openness for other insights and coping strategies, and the intake of prescribed medication. |
| K12: Crime responsibility | The extent to which the patient accepts and takes responsibility for the offences he has committed. Some reasons for the denial of responsibility are, for instance, drug- or alcohol abuse, the fact that the offence may be committed by a group of people etc. |
| K13: Coping skills | The extent to which the patient showed the correct skills to successfully resolve a confrontation with the occurrences that require adaptations (e.g., talking calmly with someone, distancing oneself from the situation, asking for help etc.). |
| K14: Labor skills | The extent to which the patient was able to properly perform work or labor activities, such as work inside the clinic, a paid job, or a voluntary position with fixed tasks and hours. |

*Note*. Reprinted from “Longitudinal network structure and changes of clinical risk and protective factors in a nationwide sample of forensic psychiatric patients” by Bogaerts, S., Spreen, M., Masthoff, E., & Jankovic, M. (2020), International Journal of Offender Therapy and Comparative Criminology, 64(15).

Table S3

An Overview of Gender Differences

| Variable | *M*(*SD*)/*N*(%) | | Test statistics |
| --- | --- | --- | --- |
|  | Males | Females |  |
| Age at admission (in years) | 32.28 (9.36) | 34.72 (9.61) | *F*(1, 814)=5.584^*^ |
| Age at discharge (in years) | 40.95 (9.50) | 42.73 (9.75) | *F*(1, 808)=2.870 |
| Treatment duration (in years) | 8.25 (3.45) | 7.59 (3.74) | *F*(1, 808)=2.924 |
| Axis I classification |  |  |  |
| Developmental disorders | 57 (7.0%) | 2 (0.2%) | χ^2^=4.048^*^ |
| Substance use disorders | 310 (38.0%) | 29 (3.6%) | χ^2^=4.685^*^ |
| Mood disorder | 55 (6.7%) | 11 (1.3%) | χ^2^=1.962 |
| Schizophrenia and other psychotic disorders | 178 (21.8%) | 21 (2.6%) | χ^2^= .192 |
| Other Axis I diagnosis | 95 (11.7%) | 20 (2.5%) | χ^2^=4.737^*^ |
| Axis II classification |  |  |  |
| Cluster A PDs | 25 (3.1%) | 2 (0.2%) | χ^2^= .443 |
| Cluster B PDs | 200 (24.5%) | 39 (4.8%) | χ^2^=8.005^*^ |
| Cluster C PDs | 22 (2.7%) | 4 (0.5%) | χ^2^= .419 |
| PD Not otherwise specified | 305 (37.4 %) | 35 (4.3%) | χ^2^= .720 |
| Multiple PDs | 18 (2.2 %) | 4 (0.5%) | χ^2^=1.025 |
| Intellectual disability | 102 (12.5 %) | 12 (1.5%) | χ^2^= .117 |
| Risk factors (range 0-4) |  |  |  |
| Psychotic symptoms | .43 (.88) | .56 (1.04) | *F*(1,770)=1.316 |
| Addiction | .48 (1.02) | .37 (.92) | *F*(1,768)=.997 |
| Impulsivity | 1.81 (1.34) | 2.03 (1.37) | *F*(1,738)=2.068 |
| Antisocial behavior | 1.35 (1.28) | 1.32 (1.19) | *F*(1,740)=.049 |
| Hostility | 1.33 (1.12) | 1.30 (1.34) | *F*(1,731)=.073 |
| Violation of terms | 1.16 (1.43) | 1.37 (1.53) | *F*(1,770)=1.828 |
| Risky network members | 1.14 (1.36) | 1.33 (1.42) | *F*(1,745)=1.484 |
| Protective factors (range 0-4) |  |  |  |
| Problem insight | 1.24 (1.00) | 1.37 (.97) | *F*(1,740)=1.327 |
| Social skills | 2.02 (.94) | 1.83 (1.08) | *F*(1,749)=3.253 |
| Self-reliance | 3.38 (.99) | 3.03 (1.23) | *F*(1,742)=8.851^*^ |
| Treatment compliance | 2.42 (1.24) | 2.46 (1.24) | *F*(1,768)=.098 |
| Taking Responsibility for index offence | 1.91 (1.28) | 2.24 (1.34) | *F*(1,665)=4.366^*^ |
| Coping skills | 1.37 (.91) | 1.16 (1.02) | *F*(1,742)=3.982^*^ |
| Labor skills | 3.08 (1.24) | 2.82 (1.35) | *F*(1,770)=2.981 |
| Criminal history (range 0-10) |  |  |  |
| Nonviolent offence | 4.43 (3.98) | 2.72 (3.64) | *F*(1,813)=15.479^**^ |
| Light/medium violent offence | 2.79 (3.12) | 1.03 (1.80) | *F*(1,812)=28.257^**^ |
| Severe violent offence | .50 (.97) | .22 (.71) | *F*(1,813)=7.313^*^ |
| Sexual offence against adults | .27 (.96) | .00 (.00) | *F*(1,813)=7.306^*^ |
| Sexual offence against minors | .16 (.96) | .11 (1.04) | *F*(1,813)= .273 |
| Arson | .25 (.78) | .43 (.83) | *F*(1,813)=4.488^**^ |
| Homicide/murder | .71 (.89) | .85 (.91) | *F*(1,813)=1.886^**^ |

Note. PD=Personality disorders. **p* < .05; ***p* < .001.
